# Ancient Remedies, Modern Medicine: A Review of Antidiabetic, Cardioprotective, and Antimicrobial Activities of Date Palm (*Phoenix dactylifera*), Tomato (*Solanum lycopersicum*), Fenugreek (*Trigonella foenum-graecum*), and Ashwagandha (*Withania somnifera*)

**DOI:** 10.3390/biology14060695

**Published:** 2025-06-13

**Authors:** Seham M. Al Raish, Razan S. Almasri, Alaa S. Bedir

**Affiliations:** 1 Department of Biology, College of Science, United Arab Emirates University, Al Ain P.O. Box 15551, United Arab Emirates; 2 Department of Nutrition, College of Medicine and Health Science, United Arab Emirates University, Al Ain P.O. Box 15551, United Arab Emirates; 201950110@uaeu.ac.ae (R.S.A.); 201950078@uaeu.ac.ae (A.S.B.)

**Keywords:** antidiabetic activity, antimicrobial properties, cardioprotective effects, medicinal plants, ethnopharmacology, bioactive compounds, phytochemicals, *Phoenix dactylifera*, *Solanum lycopersicum*, *Withania somnifera*

## Abstract

This review critically explores the pharmacological potential of four traditionally significant medicinal plants—*Phoenix dactylifera*, *Solanum lycopersicum*, *Withania somnifera*, and *Trigonella foenum-graecum*—with a specific focus on their antidiabetic, cardioprotective, and antimicrobial properties. In light of the escalating global burden of chronic metabolic diseases and the alarming rise in antimicrobial resistance, there is an urgent demand for alternative, sustainable therapeutic strategies. Drawing upon both ethnopharmacological evidence and contemporary biomedical research, this study identifies and characterizes the key bioactive constituents responsible for the observed therapeutic effects. These phytochemicals include flavonoids, phenolic acids, alkaloids, and saponins, which modulate metabolic pathways, exert antioxidative and anti-inflammatory effects, and inhibit microbial proliferation. A systematic literature search was conducted across PubMed, Web of Science, and Scopus databases, covering peer-reviewed articles published between 2000 and 2025. Inclusion criteria emphasized both in vitro and in vivo experimental models to provide a holistic understanding of molecular mechanisms and biological efficacy. Importantly, this review does not propose these plant extracts as direct substitutes for clinically established therapies but rather as potential complementary agents or sources of novel compounds for future drug development. This integrative approach underscores the relevance of traditional medicinal knowledge in guiding the discovery of plant-based therapeutics. It highlights these species as promising candidates for innovative health interventions in the context of modern biomedicine and global public health.

## Simple Summary

This article explores the potential health benefits of four traditionally recognized medicinal plants—date palm, tomato, fenugreek, and ashwagandha. These plants have been widely used in folk medicine and are now being examined in experimental studies for their possible roles in managing blood sugar, promoting heart health, and inhibiting bacterial growth. This review synthesizes current scientific findings on the bioactive compounds in these plants and highlights how they may support the development of natural therapeutic strategies for metabolic and infectious diseases. While the evidence is promising, further clinical validation is required.

## 1. Introduction

Medicinal plants (MPs) have been pivotal to both traditional and contemporary medicine globally, offering an extensive array of therapeutic benefits. The resurgence of interest in plant-based treatments has been driven by the global community’s pursuit of alternative and complementary therapies, particularly for chronic ailments such as diabetes, cardiovascular disease (CVD), and antibiotic-resistant infections [1,2]. This renewed focus aligns with a growing recognition of the limitations of conventional medicine and the potential for natural products to offer safer, more accessible therapeutic options.

In the United Arab Emirates (UAE), India, and Persian traditional medicine, MPs are celebrated not only for their medicinal value but also for their cultural significance [3,4,5]. Their utilization spans diverse therapeutic contexts, from treating metabolic disorders to addressing complex health issues like cancer, CVD, and reproductive disorders, particularly within communities in Uganda [6]. Interestingly, the application of these plants in places like Jeddah, Saudi Arabia, and Agadir, Morocco, reveals a gendered dimension to their use, where women, often the primary caregivers, exhibit a profound knowledge and reliance on MPs for family health care [7,8].

This review synthesizes current scientific literature on Phoenix dactylifera (*P. dactylifera*), *Solanum lycopersicum* (*S. lycopersicum*), *Trigonella foenum-graecum* (*T. foenum-graecum*), and *Withania somnifera* (*W. somnifera*), highlighting their established antidiabetic, cardioprotective, and antibacterial properties. Our analysis aims to underscore the pharmacological relevance of these plants in addressing urgent global health challenges. Additionally, we discuss the complexities associated with the standardization, preservation, and sustainable utilization of MPs, which are crucial for ensuring their long-term therapeutic viability [9,10].

Medicinal plants play a pivotal socio-economic and biomedical role in global health systems. According to the World Health Organization (WHO), approximately 80% of the population in developing countries rely on traditional remedies, predominantly plant-based, for primary healthcare needs [11]. This widespread usage not only sustains cultural practices but also supports rural economies and indigenous knowledge systems through the cultivation, trade, and local utilization of botanicals. Moreover, the growing consumer preference for natural health products has positioned medicinal plants as a multibillion-dollar industry with increasing demand worldwide.

In parallel, medicinal plants are emerging as valuable tools in addressing major global health challenges. The alarming rise of antimicrobial resistance (AMR) has prompted a resurgence of interest in phytochemicals with novel antimicrobial properties, which may complement or substitute synthetic antibiotics [1]. Similarly, as non-communicable diseases (NCDs) such as diabetes and cardiovascular disease become increasingly prevalent, bioactive plant compounds offer promising preventive and therapeutic benefits with fewer adverse effects compared to conventional drugs. However, the widespread exploitation of these plant resources poses risks to biodiversity and environmental sustainability, particularly where harvesting is unregulated or destructive. Sustainable cultivation practices, integration of ethnobotanical knowledge, and adherence to international conservation frameworks are urgently required to safeguard these resources for future generations [12].

Although each of the four plants—*P. dactylifera*, *S. lycopersicum*, *Trigonella foenum-graecum*, and *Withania somnifera*—have been individually studied for their pharmacological benefits, there remains a critical gap in the literature. Existing reviews tend to isolate their effects or focus on single bioactivities without comparative evaluation or mechanistic integration. Moreover, few studies have contextualized these medicinal plants within global health priorities, including metabolic diseases and antimicrobial resistance.

To address this gap, the present review systematically evaluates and compares these four plants in terms of their antidiabetic, cardioprotective, and antimicrobial properties, with particular attention to the underlying bioactive mechanisms, safety profiles, and potential for sustainable medical application.

Specifically, this study addresses the research question:

How do the bioactive compounds of these plants exert their therapeutic effects, and what is their potential for integration into modern evidence-based treatments for metabolic and infectious diseases?

Through this comprehensive review, we contribute to the expanding body of knowledge in ethnopharmacology, advocating for the development of effective, plant-based therapeutic agents that are innovative, evidence-based, and aligned with global sustainability goals.

To address this gap, the present review systematically evaluates and compares these four plants in terms of their antidiabetic, cardioprotective, and antimicrobial properties, with particular attention to the underlying bioactive mechanisms, safety profiles, and potential for sustainable medical application. Our goal is not to propose these plant extracts as standalone clinical treatments but to assess their complementary potential and identify pharmacologically active compounds that may serve as leads for novel therapeutic development, especially in contexts where integrative or adjunctive medicine is practiced.

## 2. Theoretical Framework and Literature Review

Medicinal plants have long been studied for their therapeutic applications, yet few integrative models exist to conceptualize how these species function across multiple health domains, particularly in the context of metabolic and infectious diseases. A growing body of literature supports the role of phytochemicals such as flavonoids, phenolics, alkaloids, and saponins in modulating oxidative stress, inflammatory signaling, and metabolic enzyme pathways [1,13,14].

Studies have shown that *P. dactylifera*, *S. lycopersicum*, *T. foenum-graecum*, and *Withania somnifera* each possess unique and overlapping mechanisms of action. These range from α-glucosidase inhibition and insulin sensitization in antidiabetic pathways to suppression of cardiac oxidative stress and modulation of apoptotic genes in cardioprotection [15,16,17]. Moreover, the antimicrobial effects of these species are often attributed to their membrane-disruptive phytoconstituents and immunomodulatory functions [1,18,19].

However, comparative assessments are rare. There is a gap in understanding how these plants perform side-by-side in terms of bioactivity strength, target specificity, and safety. Additionally, integrative studies exploring synergy among these plants or between plants and conventional therapies are limited. This review synthesizes current knowledge with the aim of identifying mechanistic patterns and proposing research pathways.

Humans have utilized plants and other natural products for many purposes throughout history, demonstrating a widespread knowledge, attitude, and practice toward leveraging natural environmental resources to support their well-being [20]. This reliance on natural resources extends beyond personal health and significantly influences agricultural practices [21,22]. Furthermore, medicinal plants and natural products are extensively employed due to their diverse therapeutic properties, including antimicrobial, anti-inflammatory, antioxidant, and antidiabetic effects, thus playing a crucial role in promoting health and managing various diseases [23,24,25,26].

## 3. Materials and Methods

This review rigorously examines the antidiabetic, cardioprotective, and antimicrobial properties of *P. dactylifera*, *S. lycopersicum*, *Withania somnifera*, and *Trigonella foenum-graecum*. A comprehensive literature search was conducted across four databases: PubMed, Scopus, Web of Science, and Google Scholar, employing both botanical names and synonyms to ensure the inclusion of all relevant studies. Only peer-reviewed articles published between 2000 and 2025 were considered, with a focus on the medicinal applications of these plants in managing globally prevalent diseases.

The inclusion criteria prioritized studies that provided pharmacological insights into active compounds, mechanisms of action, and therapeutic outcomes. Exclusion criteria included studies not involving medicinal use, non-peer-reviewed literature, articles published in languages other than English without reliable translation, and reviews without primary data.

This systematic review was conducted following the PRISMA 2020 guidelines. The selection criteria were stringent, prioritizing studies that provide the latest pharmacological insights into active compounds, their mechanisms of action, and clinical efficacy. Exclusions were made for non-relevant species, research not pertaining to medicinal uses, articles not subjected to peer review, and studies published in languages other than English without reliable translations.

By integrating contemporary scientific findings, this review delineates the pivotal roles these plants play in both traditional and modern medicine. It offers a comprehensive synthesis of their health benefits, particularly in managing diabetes, enhancing cardiovascular health, and mitigating microbial threats, thereby underscoring their potential to contribute to global health solutions.

### 3.1. Literature Search Strategy

A comprehensive literature search was conducted using four major databases: PubMed, Scopus, Web of Science, and Google Scholar, covering the period from 2000 to 2025. The search strategy included the botanical and common names of the plants under study (*Phoenix dactylifera*, *S. lycopersicum*, *Trigonella foenum-graecum*, and *Withania somnifera*) combined with keywords such as “antidiabetic”, “cardioprotective”, and “antimicrobial”. Boolean operators (AND/OR) were used to maximize retrieval. The search terms included both scientific and common names of the four plants, combined with pharmacological keywords such as “antidiabetic”, “cardioprotective”, and “antimicrobial”. Boolean operators (AND/OR) were used to optimize search sensitivity. The search strategy was informed by previous systematic reviews on medicinal plant bioactivity [1,2]. Only peer-reviewed articles in English were included. The review was conducted in accordance with the PRISMA 2020 guidelines [27].

### 3.2. Screening and Selection

The initial search yielded 520 records. After removing 50 duplicates, 470 unique articles remained. These were screened by title and abstract, and 380 were excluded due to irrelevance or insufficient information. The remaining 90 full-text articles were assessed for eligibility based on the following inclusion criteria:

(i) Peer-reviewed experimental studies (in vitro, in vivo, or clinical);
(ii) Focus on the therapeutic use of the specified plants;
(iii) Clear identification of plant part and extract type.

**Figure 1:**
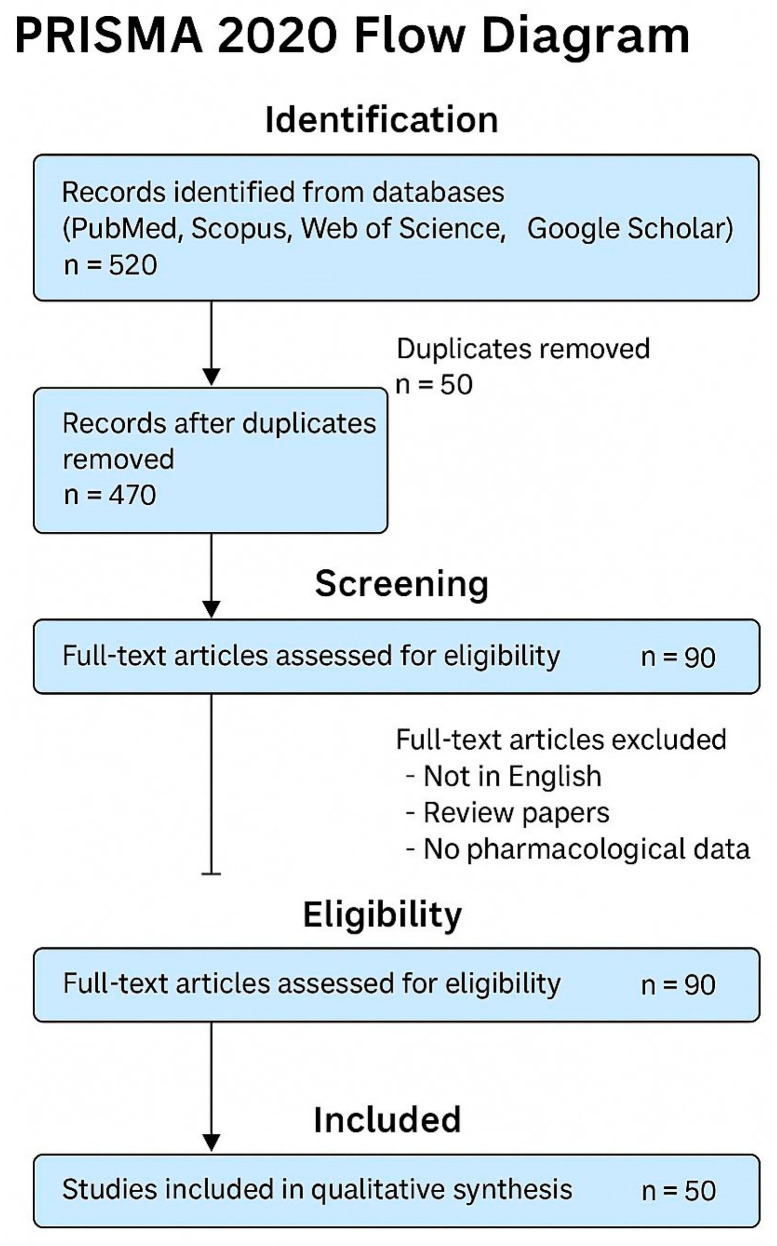
Flowchart illustrating the systematic literature screening and selection process based on PRISMA 2020 guidelines. The diagram summarizes the number of records identified, screened, excluded, and included in the final qualitative synthesis.

Articles were excluded if they were non-English, review papers, or lacked specific pharmacological data. Finally, 50 studies were included in the qualitative synthesis. The selection process is illustrated in the PRISMA flow diagram (Figure 1).

### 3.3. Data Extraction and Synthesis

For each included study, data were extracted on:

Plant species and part used;
Study type (in vitro, in vivo, or clinical);
Extract type and dosage;
Biological mechanism or molecular target;
Reported therapeutic outcome.

Studies were categorized under three main pharmacological themes: antidiabetic, cardioprotective, and antimicrobial activities. The synthesis involved narrative comparisons and tabular presentation of key findings to highlight patterns, differences in efficacy, and bioactive compound mechanisms.

## 4. Traditional Formulations and Synergistic Effects

Traditional medicinal practices frequently utilize combinations of medicinal plants rather than single-plant preparations to enhance therapeutic efficacy through synergistic interactions. Medicinal plants such as date palm (*P. dactylifera*), fenugreek (*Trigonella foenum-graecum*), tomato (*S. lycopersicum*), and ashwagandha (*Withania somnifera*) are commonly prepared in diverse formulations, including decoctions, infusions, powders, and topical preparations. These formulations may improve bioavailability, enhance efficacy, and increase safety, as the bioactive compounds within plant mixtures interact synergistically to amplify therapeutic effects and reduce potential adverse reactions [28,29,30].

### Examples of Synergistic Plant Combinations

Specific medicinal plants demonstrate significant therapeutic synergy when combined with other herbs. Fenugreek (*T. foenum-graecum*) is frequently combined with cinnamon (*Cinnamomum verum*) and bitter melon (*Momordica charantia*) to enhance antidiabetic effects by improving glycemic control and insulin sensitivity [31]. Similarly, ashwagandha is often formulated with adaptogenic herbs like holy basil (*Ocimum sanctum*) and licorice (*Glycyrrhiza glabra*) to potentiate stress relief, support adrenal function, and enhance immune responses [29,32]. These synergistic herbal combinations, rooted in traditional empirical knowledge and supported by contemporary scientific research, highlight a comprehensive therapeutic approach aiming to optimize clinical outcomes while minimizing side effects.

## 5. Medicinal Plant Usage Overview

Medicinal plants are integral to the development of therapeutic strategies for both non-communicable and communicable diseases, including cancer, cardiovascular diseases, diabetes, malaria, and tuberculosis. These plants form the backbone of healthcare systems in many regions, serving as traditional medicines that are both accessible and effective [12]. The ongoing scientific exploration of plant-based therapies highlights their critical role as sustainable natural resources with significant medicinal potential [2].

Historically, herbs have been utilized to manage and prevent a variety of ailments, ranging from abdominal pain and headaches to chronic conditions such as diabetes and hypertension. They are also employed for immune enhancement, relaxation, and cosmetic purposes, showcasing their versatility in medical applications [33,34]. In a notable historical context, ancient Egyptians leveraged a diverse array of plants such as garlic, date, lotus, and cucumber as natural remedies, utilizing entire plants or specific parts like fruits, leaves, juices, or roots. These were chosen for their rich content of bioactive compounds, including flavonoids, isoflavonoids, and alkaloids, which contribute to their therapeutic properties [35].

In the Mediterranean basin, a recognized biodiversity hotspot, efforts are ongoing among researchers and policymakers to document, conserve, evaluate, and sustainably utilize medicinal plants, thereby preserving their cultural and biological heritage [36]. Similarly, the UAE boasts a significant variety of flora, adapted to both harsh and temperate climates, providing extensive opportunities for the cultivation and application of herbal medicine within the region [37,38,39].

## 6. Medicinal Plants’ Overview of *Phoenix Dactylifera*

### 6.1. Overview

*Phoenix dactylifera*, commonly referred to as the date palm, belongs to the *Phoenix* genus within the Arecaceae family. This species, native to the arid regions of the Arabian Peninsula, North Africa, and the Middle East, has been a vital economic resource for millennia [13,40,41,42]. Adapted to harsh environments, the *P. dactylifera* is characterized by a deep root system devoid of a primary root, enabling efficient groundwater access. It flourishes across a range of altitudes and tolerates moderate salinity, often found in neglected groves or near desert water pools. The *P. dactylifera* blooms in late spring, with fruiting occurring in June and July, and is prized in urban settings for its aesthetic and ecological contributions [42,43,44].

The ripening process of dates involves several stages, Hababouk, Kimri, Khalal, Rutab, and Tamar, with the final three stages yielding the edible fruits. Khalal-stage dates are highly nutritious, though their bitter flavor is less favored in European diets compared to the sweeter, fully ripened Tamar stage [44].

*Phoenix dactylifera* are rich sources of both primary and secondary metabolites such as sugars, amino acids, and a broad spectrum of phenolic and flavonoid compounds. These contribute to its diverse pharmacological properties, including immunomodulatory, antioxidant, anti-inflammatory, and anticancer effects [13,40,45]. Traditionally, in regions like southeastern Morocco, date palms are used medicinally to manage conditions such as hypertension and diabetes [45]. They are consumed in various forms—fresh, dried, or processed [46].

Recent research has emphasized the significance of harvest timing and storage conditions on the phytochemical profiles of dates, impacting their nutritional and medicinal value [47]. Optimal pollination conditions are crucial for fruit set, with specific temperature ranges enhancing pollination success [46]. Further studies have explored the application of *P. dactylifera* in fields such as nanotechnology for antimicrobial uses and in nutrition for managing health conditions like mild anemia during pregnancy [48,49].

Comprehensive reviews have assessed the biochemical composition and biological activities of *P. dactylifera* seeds, highlighting their potential in food production, cosmetics, and medicinal supplements [50,51]. Such studies affirm the integral role of *P. dactylifera* in both traditional uses and contemporary scientific applications, spanning dietary, therapeutic, and industrial domains.

### 6.2. Antidiabetic Effect

*Phoenix dactylifera* has demonstrated significant antidiabetic potential, primarily due to its rich content of flavonoids, phenolic acids, and saponins [13,51]. These bioactive compounds contribute to glycemic regulation through mechanisms such as α-glucosidase inhibition, antioxidant enzyme activation, and reduction of oxidative stress in pancreatic and hepatic tissues [52,53]. Several in vivo studies using diabetic rodent models have reported improved blood glucose levels, insulin sensitivity, and protection of liver and kidney architecture following the administration of date seed or fruit extracts [53,54]. These outcomes support the use of *P. dactylifera* as a functional food component in diabetes management.

*Phoenix dactylifera* L. is renowned for its diverse array of bioactive compounds that significantly enhance its therapeutic potential, especially in diabetes management. The extract of *P. dactylifera* is particularly rich in flavonoids, steroids, phenols, and saponins, which play crucial roles in its antidiabetic effects [13]. Recent studies have highlighted the role of flavonoid compounds isolated from the *P. dactylifera* fruit epicarp in improving various biochemical parameters in diabetic rats, demonstrating the plant’s potential as a natural antidiabetic agent [13,52].

Moreover, studies have shown the protective effect of *P. dactylifera* seeds against diabetic complications in streptozotocin-induced diabetic rats, further validating the seeds’ efficacy in modulating oxidative stress and enhancing antioxidant enzyme activities in liver and kidney tissues. Significant reductions in biomarkers such as glucose, urea, creatinine, ALT, and AST in treated rats illustrate the potential of *P. dactylifera* seeds in early diabetic intervention [53].

Additionally, research on *P. dactylifera* seeds from various cultivars has revealed their substantial potential as skin-whitening, neuroprotective, anti-hyperglycemic, and anti-hyperlipidemic agents due to their diverse chemical compositions and bioactive profiles [54]. Furthermore, the potential of *P. dactylifera* fruits in diabetes treatment has been emphasized due to their polyphenols, which exert intense antioxidant activities and other mechanisms beneficial for managing diabetes and its complications [55].

Lastly, the nutritional and health-promoting implications of *P. dactylifera* pollen have also been studied. These studies provide insights into the pollen’s physicochemical parameters, biological activities, and nutritional values, which suggest its potential as a health supplement [56,57]. These comprehensive studies underscore the multifaceted benefits of *P. dactylifera*’s different components, reinforcing its value in both traditional and contemporary health applications (Table 1).

While several studies support its antidiabetic potential, variations in extract form, part used (seed vs. fruit), and dose complicate direct comparisons, as further examined in Section 7.

### 6.3. Cardioprotective Effect

*Phoenix dactylifera* is a valuable source of bioactive compounds recognized for their cardioprotective effects. The extracts of this plant are rich in phenolic acids, such as hydroxybenzoic and hydroxycinnamic acids, and a range of flavonoids, including flavonoid glycosides, catechin flavanol, and anthocyanidins, which have been shown to exhibit significant antioxidant activities [58]. Research by Alhaider et al. (2017) and others highlights the high concentrations of these phenolic and flavonoid compounds in *P. dactylifera* fruit extracts, which are linked to their potent antioxidant properties [59]. These compounds contribute markedly to cardiovascular health, showing effects such as improved cardiac biomarkers and protection against myocardial infarctions in various studies [58].

The antioxidant properties of *P. dactylifera* are notably linked to substantial protective effects against induced myocardial infarctions in vivo, underlining its potential as a natural source of cardioprotective agents. Studies by Alqarni et al. (2019) have explored the in vivo antioxidant and antihyperlipidemic potential of Ajwa date polyphenol extract, demonstrating significant regulatory impacts on body weight, lipid profiles, and cardiac markers in rats fed a cholesterol-rich diet [60]. Additionally, the cardioprotective role of *P. dactylifera* was further evidenced by Al-Yahya et al. (2016), who investigated the effects of Ajwa date extract in attenuating cardiomyopathy through the modulation of oxidative, inflammatory, and apoptotic pathways in a rodent model [61].

Furthermore, Al-Jaouni et al. (2019) found that the Ajwa nano preparation effectively shields against doxorubicin-associated cardiotoxicity, highlighting its role in preventing cardiac ischemia and enhancing cardiac antioxidant capacity [62]. This spectrum of research demonstrated that *P. dactylifera* not only provides direct cardioprotective effects but also engages multiple biochemical pathways that support cardiovascular health, making it a promising candidate for natural health interventions against heart disease [62].

In the context of cardiovascular health, the cardioprotective effects of *P. dactylifera* extracts described in this review could primarily serve as supportive or adjunctive therapies rather than standalone treatments. Specifically, extracts from date palm fruits might have clinical relevance for alleviating cardiotoxic side effects associated with anthracycline chemotherapy, such as doxorubicin, and for complementing conventional treatments of hypertension. The potential mechanisms include reducing oxidative stress, modulating inflammation, and improving endothelial function. Nevertheless, these plant-derived compounds should not be viewed as replacements for existing pharmaceutical therapies. Comprehensive clinical studies remain necessary to confirm their safety, efficacy, dosage, and potential interactions with standard cardiovascular medications (Table 1) [61,62].

### 6.4. Antibacterial Effect

The four medicinal plants discussed exhibit notable antibacterial properties primarily attributed to bioactive compounds such as flavonoids, alkaloids, and saponins, which have demonstrated efficacy against a variety of pathogenic bacteria. *S. lycopersicum* extracts, particularly rich in polyphenolic compounds and flavonoids, have shown inhibitory activity against *Staphylococcus aureus* and *Escherichia coli* [63]. *T. foenum-graecum* contains trigonelline and saponins, compounds associated with significant antibacterial action against both Gram-positive and Gram-negative bacteria, including resistant strains [64]. Similarly, the antibacterial activity of *W. somnifera* is primarily due to withanolides and alkaloids that disrupt bacterial cell membranes and inhibit bacterial growth [18,19]. Lastly, *P. dactylifera* seeds exhibit antimicrobial properties linked to phenolic compounds and flavonoids, which are effective against common pathogens [65]. Collectively, these findings highlight the promising role of these plants as natural antibacterial agents, warranting further investigation into their potential clinical application. Clinical studies involving date palm consumption have indicated beneficial effects on glycemic control. Alkaabi et al. (2011) reported improved glycemic response among diabetic patients consuming specific date varieties [66]. However, further comprehensive clinical trials are necessary to establish clear dosage recommendations and validate therapeutic effectiveness (Table 1) [66,67].

**Table 1:** Antidiabetic, cardioprotective, and antibacterial effects of *Phoenix dactylifera* extracts in various experimental models.

**Antidiabetic Effect**						
Scientific name	Trial type	Model or method	Used Part or form	Dose and duration	Therapeutic effect	Reference
*P. dactylifera*	In vivo	Male Wistar albino rats male aged 2–3 weeks	Methanolic Extract of *P. dactylifera*	5 mg/kg/day for 25 days	Improve glucolipid balance Improve oxidative stress Confer both direct and indirect cardioprotective effects	[68]
	In vitro	Biochemical assays	Seeds	-	Antioxidant, antimicrobial, and anti-inflammatory properties	[51]
	In vivo	Diabetic rat model	Extract from epicarp or seeds	-	Improved biochemical parameters (e.g., glucose, urea, creatinine), reduced ALT and AST levels, enhanced antioxidant enzyme activity, modulated oxidative stress	[13,52]
	In vivo	Male Wistar rats weighing 200–250 g	The aqueous suspension of *P. dactylifera* seeds (aqPDS)	1 g/kg/d for 4 weeks.	Ameliorate glucolipid imbalance, reduce oxidative stress, and protect liver and kidney architecture	[53]
	In vitro	Enzyme inhibition assays	Date seed extracts	-	Inhibition of α-glucosidase and α-amylase, antioxidant activity	[54]
	In vitro	Enzyme inhibition assays	Date fruit extract	-	Inhibition of α-glucosidase, α-amylase; improvement in glucose tolerance	[55]
	In vitro	Enzyme inhibition assays	DP pollen	-	Antioxidant properties, enzyme inhibition (α-glucosidase, α-amylase)	[56,57]
**Cardioprotective**						
	In vivo	Male Wistar albino rats male aged 2–3 weeks	Methanolic Extract of *P.Dactylifera*	5 mg/kg/day for 25 days	Improve glucolipid balance Improve oxidative stress Confer both direct and indirect cardioprotective effects	[68]
	In vivo	Hypercholestrolemic rats	Polyphenol-rich extract	25, 50, and 100 mg/kg.bw	Significant antioxidant effects, improved lipid profile, and reduced cardiac markers in cholesterol-fed rats	[60]
	In vivo	Rats	Ajwa date extract	250 and 500 mg/kg.bw	Cardioprotection via modulation of oxidative stress, inflammation, and apoptosis pathways	[61]
	In vivo	Twenty-four male Wistar rats (200–250 g)	Nano-preparation of Ajwa date extract	1.4 g/kg orally 1 h before doxorubicin infusion	Protection against cardiotoxicity, prevention of ischemia, and enhancement of antioxidant capacity	[62]
	In vitro	Antioxidant assays	Phenolic and flavonoid extracts from fruits	-	High antioxidant activities due to phenolic acids and flavonoid glycosides contributing to cardiovascular health	[58,59]
**Antibacterial**						
	In vitro	-	DP extracts	-	Pseudomonas aeruginosa cell walls were damaged	[69]
	In vitro	Antibacterial testing against *E. coli*, *B. cereus*, *S. aureus*, and *Serratia marcescens*	Methanolic extract of Ajwa dates	-	Significant antibacterial activity against a range of bacteria	[70]
	In vitro	Investigation of cell wall damage in *P. aeruginosa*	Flavonoid glycosides in DP extracts	-	Damage to the cell walls of *P. aeruginosa* causing pore formation in the bacterial cells	[69]
	In vivo and in vitro	Health benefits testing (e.g., oxidative stress reduction, hepatoprotective effects)	Aqueous and mixed aqueous/organic extracts	-	Exhibited oxidative stress reduction, free radical scavenging, coronary heart disease prevention, hepatoprotective effects, anti-inflammatory, and anticancer activities	[70]
	In vitro	Bioactive compound analysis	Ethanol extract of Ajwa dates	-	Identified 33 active components, including flavonoid glycosides, phenolic acid derivatives, fatty acids, and lignans, which contribute to therapeutic and health-promoting properties	[69]

*P. dactylifera: Phoenix dactylifera*, ALT: Alanine Aminotransferase, AST: Aspartate Aminotransferase, aqPDS: Aqueous Suspension of *Phoenix dactylifera* Seeds, mg/kg.bw: Milligrams per Kilogram of Body Weight. *P. aeruginosa: Pseudomonas aeruginosa*. DP: Date palm.

## 7. *Solanum lycopersicum*

### 7.1. Overview

*Solanum lycopersicum* (tomato) is widely recognized for its valuable bioactive compounds, such as lycopene, flavonoids, phenolic acids, and vitamins [16]. These phytochemicals have shown significant therapeutic potential related to diabetes management, cardiovascular health, and antibacterial activity [63,71]. Lycopene, in particular, has demonstrated the ability to reduce oxidative stress, a key factor involved in the development of diabetes and cardiovascular complications [72]. Despite these promising outcomes observed in preclinical studies, further clinical research is necessary to confirm the effectiveness and optimal applications of tomato-derived bioactive compounds in human health. Furthermore, tomatoes are utilized globally beyond their common culinary applications. In traditional African medicine, tomato leaves and fruits are used for their antimicrobial and wound-healing properties [73]. In South America, tomatoes are traditionally applied for treating skin disorders and gastrointestinal conditions due to their anti-inflammatory and antioxidant effects [74]. Additionally, in East Asian traditional medicine, especially in China and Korea, tomato consumption is valued for cardiovascular health benefits attributed to its high lycopene content, and extracts are employed for promoting general health and longevity [75,76,77,78]. This diverse global application highlights the universal therapeutic value of tomatoes across various cultures.

### 7.2. Antidiabetic Effect

*Solanum lycopersicum* (tomato) contains a diverse array of bioactive compounds, prominently lycopene, which have attracted significant attention due to their potential role in diabetes management. Lycopene, a potent lipid-soluble carotenoid antioxidant abundant in tomatoes, has shown notable preclinical efficacy in improving glucose metabolism, insulin sensitivity, and reducing oxidative stress, which are critical aspects in diabetes pathophysiology [71,72,79]. Experimental studies in animal models, particularly diabetic rodents, have demonstrated that dietary supplementation with lycopene-rich tomato extracts can effectively lower blood glucose levels, enhance insulin sensitivity, and modulate biomarkers of diabetes-induced oxidative damage and inflammation [79,80,81]. For example, Ali and Agha (2009) observed significant amelioration of diabetes-induced dyslipidemia and oxidative stress markers following the administration of tomato-derived lycopene in diabetic rats [71].

Despite these promising preclinical findings, human clinical studies have yielded inconsistent results regarding tomato’s antidiabetic effects. Some small-scale studies suggest modest benefits of lycopene supplementation or tomato product consumption, showing minor reductions in fasting glucose levels and oxidative stress markers in diabetic patients [81,82]. However, other well-designed clinical trials found negligible or no significant impact on critical diabetes parameters such as fasting blood glucose (FBG), hemoglobin A1c (HbA1c), or insulin resistance markers following dietary or supplemental intake of lycopene-rich tomato products [83]. These contrasting outcomes underline the challenges in translating promising preclinical results into clinically significant therapeutic strategies. Such discrepancies could be attributed to variability in the study populations, dosages of lycopene used, duration of treatment, or differences in the formulation and bioavailability of lycopene. Limited clinical data suggest lycopene intake is inversely associated with disturbed glucose metabolism and reduced risk of type 2 diabetes, but larger, standardized trials are required to establish definitive clinical recommendations [84].

Consequently, although tomato-derived lycopene demonstrates clear potential in animal studies, current evidence from human clinical trials remains insufficient to conclusively establish its role in diabetes management. Thus, rigorous, larger-scale and longer-duration human studies are critically needed to validate these preclinical findings, define optimal therapeutic dosages, and clarify the specific populations that might benefit most significantly from lycopene supplementation or dietary interventions based on tomato-derived products (Table 2).

However, the observed hypoglycemic effect of lycopene varies across studies, especially between animal and human trials, as discussed in Section 7.

### 7.3. Cardioprotective Effect

**Figure 2:**
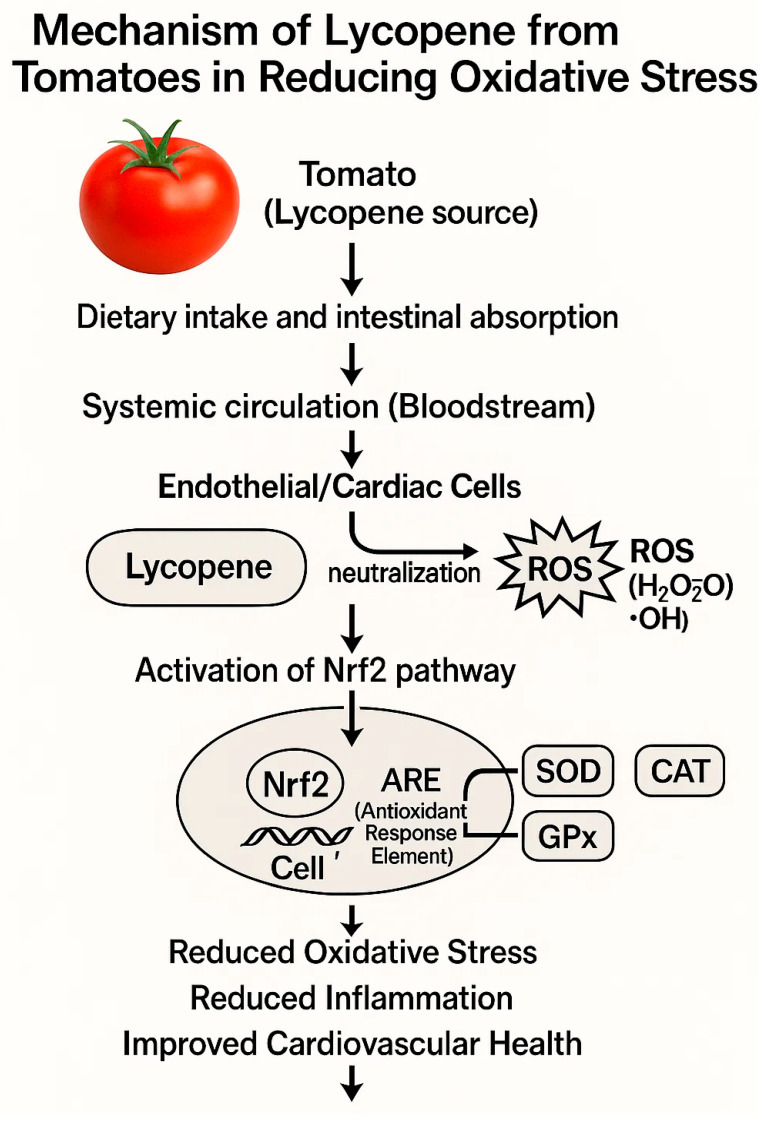
iMechanism illustrating how lycopene from tomatoes reduces oxidative stress. Lycopene, following dietary absorption and systemic distribution, enters target cells where it neutralizes reactive oxygen species (ROS). Additionally, lycopene activates the Nrf2 pathway, leading to the upregulation of antioxidant enzymes (SOD, CAT, GPx) via binding to the antioxidant response element (ARE) within the cell nucleus. Ultimately, this mechanism results in reduced oxidative stress, decreased inflammation, and improved cardiovascular health.

Lycopene-rich diets, typically including foods such as tomatoes, watermelon, grapefruit, and papaya, have been studied for their potential benefits in reducing cardiovascular risk factors. Lycopene is a potent antioxidant known to mitigate oxidative stress, reduce inflammation, and enhance endothelial function, all of which play crucial roles in preventing cardiovascular disease (CVD) [83,84]. Epidemiological studies and clinical trials suggest an inverse relationship between dietary lycopene intake and the incidence of cardiovascular diseases. For instance, lycopene supplementation has demonstrated improvements in endothelial function, reduction in LDL cholesterol oxidation, and decreased inflammation markers, which are strongly associated with lower cardiovascular risk [76,84,85]. However, clinical evidence varies, with some studies showing modest cardiovascular benefits and others indicating limited or no significant effect. Thus, while there is promising evidence supporting the cardiovascular benefits of lycopene-rich diets, definitive conclusions require further large-scale randomized controlled trials to clearly establish their effectiveness in clinical practice. Clinical trials on tomato-derived lycopene supplements suggest potential cardiovascular benefits, including improved blood pressure and lipid profiles. A systematic review and meta-analysis by Cheng et al. (2017) highlighted these cardiovascular improvements, although results across studies have varied, emphasizing the need for more consistent, large-scale clinical trials (Table 2) (Figure 2) [76,84]. 

### 7.4. Antibacterial Effect

*Solanum lycopersicum* contains bioactive compounds with various biological activities. Research has highlighted the importance of tomato lectins (TCLs), focusing on their biological functions rather than purification, as their physiological roles remain inadequately understood despite extensive historical investigation [63]. The previously reported inhibited growth rates (6.84% after 8 h and 0.51% after 16 h of treatment) represent the percentage reduction of bacterial proliferation relative to untreated controls at specific incubation periods [63]. These effects were assessed through direct incubation of bacterial cultures with tomato extracts under controlled laboratory conditions, ensuring continuous exposure at approximately 37 °C [63]. Additionally, the term ‘ozone microbubble water’ refers to water infused with extremely fine ozone bubbles, enhancing antimicrobial efficacy. This treatment method is applied externally to disinfect tomatoes and is not derived from tomato constituents [86].

Recent studies further expand our understanding of the antimicrobial capabilities of tomatoes. Tam et al. (2021) reported that natural powders derived from various parts of *S. lycopersicum* plants, particularly those containing tomatine, significantly inhibited the growth of pathogenic protozoa, bacteria, and fungi [87]. Moreover, Hou et al. (2022) demonstrated that ozone microbubble water effectively inactivated bacteria on *S. lycopersicum* without affecting their physical and sensory characteristics [86]. Silva-Beltrán et al. (2015) found that *S. lycopersicum* plant extracts, especially from leaves, exhibited strong antimicrobial and antioxidant activities, correlating positively with their phenolic, flavonoid, and chlorophyll contents [88]. Farvardin et al. (2023) highlighted the potent antimicrobial properties of *S. lycopersicum* heme-binding protein 2 against a range of pathogens [89]. Lastly, Kwon et al. (2024) discovered tomato-derived antimicrobial peptides with significant effectiveness against *typhoidal Salmonella*, offering insights into potential public health strategies using tomato and its derivatives (Table 2) [90].

**Table 2:** Antidiabetic, cardioprotective, and antibacterial effects of *Solanum lycopersicum* extracts in various experiments.

**Antidiabetic**						
Scientific name	Trial type	Model or method	Used Part or form	Dose and duration	Therapeutic effect	Reference
*S. lycopersicum*	In vivo	Hypertension patients	Antioxidant-rich tomato juice extract	250 mg/day for eight weeks	Systolic blood pressure decreased Diastolic blood pressure decreased	[91]
	In vivo	Diabatic patient	Serum lycopene	-	Inverse association between lycopene levels and disturbed glucose metabolism or T2DM; decrease in fasting glucose observed	[16]
	In vivo	Normal rate	Lycopene-rich tomato homogenates	Daily for 4 weeks	Improved glucose tolerance and enhanced insulin sensitivity	[72]
	In vivo	Hyperglycemic rats	Lycopene (exogenous administration)	90 mg/kg body weight	Reduced glucose and H_2_O_2_ levels, improved total antioxidant status, and normalized lipid profiles, demonstrating significant hypoglycemic, hypolipidemic, and antioxidant effects	[71]
	In vitro	Tomato, bioactive compounds	Lycopene	-	Antidiabetic effects via antioxidant, anti-inflammatory actions	[80,81]
	In vivo	Thirty-two type 2 diabetes patients	Raw tomato	200 g/day for 8 weeks	Suggested cardiovascular risk reduction in T2DM patients	[82]
**Cardioprotective**						
	In vivo	Hypertension patients	Antioxidant-rich tomato juice extract	250 mg/day, for 8 weeks	Systolic blood pressure decreased Diastolic blood pressure decreased	[91]
	In vivo	Human dietary intervention studies	Lycopene-rich diets	Long-term dietary consumption	Reduction in systolic and diastolic blood pressure, improved HDL levels, decreased CRP (inflammation marker)	[16,92]
	In vitro	Antioxidant and anti-inflammatory assays	Tomato	-	Antioxidant and antiplatelet effects, showcasing potential for functional food development	[93]
	In vitro	Bioavailability and metabolic studies	Tomato carotenoids and phenolic extracts	-	Increased antioxidant capacity and modulation of oxidative stress pathways	[94]
**Antibacterial**						
	In vitro	-		-	Ability to inhibit the growth of certain bacteria such as *S. boydii*. Similarly, *S. dysenteriae* exhibited time-dependent inhibition rates of 5.17%, 3.67%, and 2.85% under the same conditions. In the case of Staphylococcus aureus, TCLs inhibited growth at rates of 6.84% and 0.51% after 8 and 16 h of treatment, though their activity diminished after 24 h. Conversely, *E. coli* showed mitogenic growth (7–9%) rather than inhibition when treated with TCLs	[95]
	In vitro	Composition analysis and antimicrobial testing	Processing waste of ten tomato varieties	-	Moderate correlation between antimicrobial activity against *S. aureus* and *isochlorogenic* acid content; potential bioactive uses of tomato waste	[96]
	In vitro	Antimicrobial testing on pathogenic protozoa, bacteria, fungi	Natural tomato powders with tomatine	-	Significant inhibition of pathogens due to tomatine content	[87]
	In vitro	Ozone microbubble water treatment on tomatoes	Whole tomatoes	-	Effective bacterial inactivation without altering physical/sensory characteristics of tomatoes	[86]
	In vitro	Antimicrobial and antioxidant analysis	Tomato plant extracts (leaves)	-	Strong antimicrobial and antioxidant activities correlated with phenolic, flavonoid, and chlorophyll contents	[88]
	In vitro	Antimicrobial assay on pathogens	Tomato heme-binding protein 2	-	Potent antimicrobial properties against a wide range of pathogens	[89]

*S. lycopersicum: Solanum lycopersicum*, T2DM: Type 2 Diabetes mellitus, CRP: C-reactive protein, HDL: High-density lipoprotein, H_2_O_2_: Hydrogen peroxide, TCLs: Tomato carotenoid lipids, mg/kg.bw: Milligrams per kilogram of body weight.

## 8. *Trigonella foenum-graecum*

### 8.1. Overview

*Trigonella foenum-graecum*, commonly known as fenugreek, is an annual herb classified within the genus *Trigonella* and the family Fabaceae (Leguminosae) [97]. It is native to the Indian subcontinent and the Eastern Mediterranean; the plant is commonly grown in regions of Australia, North America, Africa, central Europe, and Asia [4]. It grows to a height of 0.3 to 0.8 m and features long, slender stems adorned with trifoliate, gray-green toothed leaves 20–25 mm long. The plant produces white to yellow flowers that bloom from June to July [98]. *T. foenum-graecum* seeds have long been esteemed for their therapeutic properties across various cultures, providing numerous health benefits due to their rich nutritional composition and bioactive constituents [99,100]. The bitter taste of *T. foenum-graecum* seeds is attributed to their alkaloid and oil constituents, which are absent in defatted seeds [97].

*Trigonella foenum-graecum* has traditionally been utilized to address and prevent health conditions such as diabetes, hypercholesterolemia, and *E. coli* infections [101]. Its therapeutic effects are mainly due to its phytoconstituents, including saponins, galactomannans, trigonelline, 4-hydroxyisoleucine, and flavonoids like apigenin derivatives [101]. The dietary fiber in fenugreek seeds significantly reduces blood glucose and cholesterol levels, contributing to its potential as a functional food [102]. The seeds and leaves of *T. foenum-graecum* also contain numerous bioactive compounds with metabolic benefits, including anti-inflammatory, antioxidative, antihyperlipidemic, and anticarcinogenic properties, which support its use in functional food development [103].

*Trigonella foenum-graecum*’s adaptability to diverse growing conditions, including marginal lands, and its moderate tolerance to drought and salinity make it a valuable crop for various agricultural systems. It is also suitable for heavy metal remediation and serves as an off-season fodder crop [104]. The seeds’ hydrocolloids, particularly *T. foenum-graecum* seed gum, exhibit excellent emulsification, thickening, and packaging properties, with demonstrated antioxidant and antifungal activities, making them suitable for food, pharmaceutical, and cosmetic industries [105]. Moreover, genetic and biochemical studies on *T. foenum-graecum* have identified stable genotypes with high yields and bioactive compound content, supporting their potential for consistent medicinal and nutritional applications [106].

### 8.2. Antidiabetic Effect

*Trigonella foenum-graecum* (fenugreek) is widely recognized for its significant antidiabetic properties, primarily attributed to bioactive compounds such as saponins, galactomannans, trigonelline, and notably 4-hydroxyisoleucine [102,106,107]. These compounds effectively improve glucose metabolism, enhance insulin secretion, and reduce blood glucose levels in diabetic models [106,108]. Clinical studies demonstrate that regular oral administration of fenugreek seed powder significantly improves lipid profiles and insulin sensitivity in patients with type 2 diabetes, showing clear reductions in fasting blood glucose and glycated hemoglobin (HbA1c) levels [109]. Additionally, polyphenols such as rhaponticin and isovitexin present in fenugreek have been shown to modulate glucose absorption in intestinal cells and enhance pancreatic β-cell function, further reinforcing fenugreek’s therapeutic potential in managing diabetes and insulin resistance [108,110]. The alkaloid trigonelline also shows beneficial hypoglycemic effects and contributes to fenugreek’s overall antidiabetic activity [111]. Fenugreek has demonstrated clear benefits in glycemic control in multiple clinical studies. A notable multicenter clinical trial showed that fenugreek seed extract significantly improved fasting blood glucose and glycemic control in patients with type 2 diabetes [112]. Further standardized studies could reinforce these findings and refine therapeutic guidelines. These findings collectively affirm the robust antidiabetic efficacy of *T. foenum-graecum* and its bioactive components, supporting its use as a viable natural therapy for diabetes management and related metabolic disorders (Table 3).

### 8.3. Cardioprotective Effect

*Trigonella foenum-graecum* exhibits notable diabetic cardioprotective properties through multiple mechanisms. Recent studies highlight its potential in addressing cardiomyopathy and other cardiovascular conditions by improving metabolic abnormalities and oxidative stress and by modulating apoptotic gene expression [113]. Notably, in vitro studies reveal that *T. foenum-graecum* at a concentration of 160 µg/mL can safeguard neonatal rat cardiomyocytes against CoCl_2_-induced hypoxia, enhance calcium signaling and beating rate, and beneficially alter gene expression, underscoring its therapeutic potential against hypoxia-induced cardiac injuries [114].

Further investigations have confirmed *T. foenum-graecum*’s at cholesterol-lowering capabilities, attributable to its rich composition of saponins, diosgenin, galactomannan, coumarin, nicotinic acid, sapogenins, scopoletin, and trigonelline. These components exhibit various pharmacological effects, including enhancing the activity of antioxidant enzymes like SOD, CAT, and GPx, and reduced GSH in rat models of isoproterenol-induced myocardial infarction, thereby asserting *T. foenum-graecum*’s at cardioprotective actions [107].

In models of streptozotocin-induced diabetic rats, *T. foenum-graecum* at seed extract has demonstrated promising effects in treating diabetic cardiomyopathy by influencing genes associated with apoptosis, such as downregulating the pro-apoptotic Bax gene and upregulating the anti-apoptotic B-cell lymphoma 2 gene [113]. Additionally, dietary interventions with *T. foenum-graecum* at seeds in rats with experimentally induced myocardial infarction have shown improvements in pathological changes in cardiac tissue and lipid profiles in both blood and cardiac samples, highlighting its efficacy in hypercholesterolemic conditions (Table 3) [97,107].

Despite promising antioxidant and lipid-lowering properties, some studies show inconsistent cardioprotective effects, likely due to variability in dosage and model systems, as analyzed in Section 7.

### 8.4. Antibacterial Effect

*Trigonella foenum-graecum* seeds have demonstrated significant antibacterial and antimicrobial properties, evidenced by various studies on their extracts and composites. Ethanol extracts from *T. foenum-graecum* seeds exhibit strong antibacterial activities against pathogens like *S. aureus* and *P. aeruginosa*, with naringenin also showing antifungal and antimicrobial effects [64,115]. Additionally, *T. foenum-graecum* has been used in nanocomposites with silver nanoparticles, as reported by Sitohy et al. (2021), which highlighted the potential of these composites to inhibit pathogenic bacteria effectively [116]. These composites were synthesized by immobilizing *T. foenum-graecum* seed proteins with silver nanoparticles, demonstrating enhanced inhibitory activity compared to the nanoparticles or proteins alone [116]. Furthermore, studies by Hadi and Mariod (2022), and Sindhusha and Rajasekar (2023) have also underscored *T. foenum-graecum*’s capability in reducing biofilm activities and its effectiveness against multidrug-resistant strains, providing a novel approach to combatting antibiotic resistance [117,118]. These diverse studies collectively underline the potential of *T. foenum-graecum* not only in traditional applications but also in modern medical and therapeutic fields.

It is important to acknowledge that while in vitro studies demonstrate initial evidence of antimicrobial properties, they do not necessarily predict clinical efficacy. Killing microorganisms under controlled laboratory conditions has limitations because these conditions differ significantly from complex in vivo environments. In vitro assays primarily serve as an essential preliminary step to identify and characterize bioactive compounds and elucidate possible antimicrobial mechanisms. Therefore, further in vivo and clinical studies are crucial to confirm the practical therapeutic efficacy and safety of these compounds in real-world applications (Table 3) [1,2].

**Table 3:** Therapeutic potential of *Trigonella foenum-graecum*: antidiabetic, cardioprotective, and antibacterial effects in various experiments.

**Antidiabetic**						
Scientific name	Trial type	Model or method	Used Part or form	Dose and duration	Therapeutic effect	Reference
*T. foenum-graecum*	In vivo	Young Wistar strain albino rats	Trigonelline is a bioactive compound of *T. foenum-graecum*	30 days	Protected cardiac tissue from alcohol-induced toxicity	[108]
	In vivo	Diabetic rats	Fenugreek seeds, leaves	-	Reduction of blood glucose levels	[101]
	In vivo	-	Fenugreek	-	Antidiabetic, antihyperlipidemic, anti-obesity, anticancer, anti-inflammatory, antioxidant, antibacterial	[64,69,110,111]
	In vivo	-	Fenugreek seeds and compounds	-	Improves blood glucose, insulin resistance, insulin sensitivity, and lipid profiles	[15]
	In vivo	Diabetic patients	Seed powder solution	25 g orally twice a day for one month	Improving lipid metabolism in type II diabetic patients with no adverse effects	[109]
**Cardioprotective**						
		Young Wistar strain albino rats	Trigonelline is a bioactive compound of *T. foenum-graecum*	30 days	Protected cardiac tissue from alcohol-induced toxicity	[108]
	In vivo	Neonatal rat cardiomyocytes, CoCl_2_-induced hypoxia	Fenugreek	Single application	Protection against hypoxia, improved calcium signaling, increased cardiomyocyte	[114]
	In vivo	Rat model	Fenugreek seed extract	-	Improved antioxidant enzyme activity, reduced oxidative stress, and amelioration of cardiac damage	[107]
	In vivo	Streptozotocin-induced diabetic rats	Fenugreek seed extract	-	Improved diabetic cardiomyopathy markers	[113]
	In vivo	Hypercholesterolemic rat model	Fenugreek seeds (dietary intervention)	-	Enhanced lipid profiles, reduced pathological changes in cardiac tissue, and cardioprotection in hypercholesterolemic conditions	[97,107]
	In vivo	Neonatal rat cardiomyocytes, CoCl_2_-induced hypoxia	Fenugreek	Single application	Protection against hypoxia, improved calcium signaling, increased cardiomyocyte	[114]
**Antibacterial**						
	In vitro	Wistar rat model	Fenugreek extract alone and in combination with Bifidobacterium breve	Treated orally at the same time once daily for 2 weeks	Inhibition of *H. pylori* growth	[119]
	In vitro	Antibacterial activity assay	Methanolic extract	Not specified	Significant antibacterial activity against *E. coli*, *B. cereus*, *S. aureus*, and *Serratia marcescens*	[70]
	In vitro	Structural analysis on *P. aeruginosa*	Flavonoid glycosides from extracts	Not specified	This caused structural damage to *P. aeruginosa* cell walls, leading to pore formation and showcasing strong antimicrobial potential	[69]

*T. foenum-graecum*: *Trigonella foenum-graecum*, *E. coli*: *Escherichia coli*, *B. cereus*: *Bacillus cereus*, *S. aureus*: *Staphylococcus aureus*, *P. aeruginosa*: *Pseudomonas aeruginosa*, *H. pylori*: *Helicobacter pylori*. CoCl_2_: Carbon dichloride oxide.

## 9. Medicinal Plants’ Overview of *Withania somnifera*

### 9.1. Overview

*Withania somnifera* is a MP that has been used for more than 3000 years in Ayurvedic and indigenous medicine; commonly known as ashwagandha, it is a perennial shrub within the genus *Withania* of the Solanaceae family [14,120]. It is found in the drier regions of South Africa, Egypt, Morocco, Jordan, Afghanistan, Sri Lanka, Pakistan, Baluchistan, and India. It is extensively cultivated in the Indian states of Madhya Pradesh, Uttar Pradesh, Punjab’s plains, and the northwest regions of Gujarat and Rajasthan [121]. It is a woody, xerophytic, evergreen shrub that reaches a height of roughly two meters and a width of one meter. Its fruit is a round, hairless berry encased in a membrane calyx. When mature, it turns orange-red and has many kidney-shaped, pale brown seeds inside. Strong and meaty, the roots have an acidic, bitter taste and a pungent smell [122]. The plant blooms year-round, with peak flowering from March to July [123].

This versatile MP has garnered attention for its diverse pharmacological properties, with clinical and preclinical trials confirming its efficacy in treating conditions such as hepatotoxicity, neurological disorders, anxiety, Parkinson’s disease, and hyperlipidemia [14,124]. Phytochemical studies have highlighted a variety of bioactive compounds in *W. somnifera*, notably steroidal lactones known as withanolides, which contribute significantly to their medicinal value [125]. The plant’s roots contain a notable concentration of the alkaloid withanine, comprising a substantial part of its alkaloid profile, along with steroids, saponins, phenolics, flavonoids, and glycosides [122]. Both the fruits and leaves of the plant have been found to contain functional components, with the leaves also displaying insect-repellent properties.

Historically, *W. somnifera* has been incorporated into medicinal formulations for its antipyretic, analgesic, adaptogenic, antidiabetic, and anti-inflammatory effects, emphasizing its role in both traditional and contemporary therapeutic practices [126]. Additionally, recent advancements in extraction techniques and their inclusion in food products such as baked goods, juices, and dairy items underscore its growing relevance in nutraceutical and functional food applications [125,127]. Comparative studies of in vitro cultured and field-grown *W. somnifera* roots have also shown varying levels of free radical scavenging activity and metabolic profiles, suggesting potential tailored uses in medicinal and functional food products [128].

### 9.2. Antidiabetic Effect

*Withania somnifera* has demonstrated promising antidiabetic properties across various studies. Preclinical research highlights its ability to significantly lower blood glucose levels in animal models, primarily through the actions of key compounds like *W. somnifera*, which modulates critical pathways such as Nrf2/NFκB signaling in type 1 diabetes models [126,129]. While clinical studies present mixed results regarding its direct blood glucose-lowering effects, consistent findings support its beneficial impact on lipid profiles, body weight, and blood pressure in diabetic patients [126,130,131].

*Withania somnifera*’s antidiabetic mechanism involves multiple biological pathways. Notably, anti-adipogenic withanolides from the root inhibit the differentiation of preadipocytes into adipocytes, addressing obesity-related metabolic disorders [17]. Furthermore, *W. somnifera* extract has been shown to enhance energy expenditure by improving mitochondrial function in adipose tissue and skeletal muscle, further supporting its therapeutic potential [17].

Supporting clinical studies have confirmed *W. somnifera*’s antidiabetic potential. Administration of its root powder significantly lowered blood glucose levels in hypercholesterolemic and diabetic patients, comparable to traditional oral hypoglycemic drugs [14,122]. Moreover, aqueous extracts of *W. somnifera* effectively reduced blood glucose, HbA1c, and insulin levels in animal models, with both root and leaf extracts improving glucose uptake in various tissues [122].

Long-term administration of these extracts in diabetic rat models normalized critical diabetes markers such as glucose levels, tissue glycogen, and glucose-6-phosphatase activities. The antidiabetic activity is attributed to the presence of phenolics and flavonoids, and notably, *Withaferin* also blocks inflammatory responses in pancreatic tissues and shows anti-glycating effects [122].

These comprehensive findings collectively suggest that *W. somnifera* holds significant promise as a natural antidiabetic agent. It acts through multiple mechanisms to improve glucose metabolism, lipid profiles, and overall metabolic health in diabetic conditions (Table 4).

### 9.3. Cardioprotective Effect

*Withania somnifera*, recognized for its wide-ranging medicinal benefits, has garnered attention for its potent cardioprotective properties. Recent studies, such as those by Wiciński et al. (2024) [17] and Mikulska et al. (2023) [126], highlight its efficacy in reducing myocardial necrosis and oxidative stress in cardiac tissue through mechanisms involving the upregulation of mitochondrial anti-apoptotic pathways and enhancement of the Bcl-2/Bax ratio. Additionally, these studies mentioned that lower doses of withaferin A have demonstrated greater effectiveness in these respects [17,126]. Further, Paul et al. (2021) explored its role in mitigating doxorubicin-induced cardiotoxicity, underscoring its potential in managing drug-induced cardiac damage [122]. Behl et al. (2020) discuss the broader pharmacological profile of withaferin A, detailing its multi-targeted approach to disease management, including cancer and neurodegenerative diseases, by modulating various molecular pathways such as Peroxisome Proliferator-Activated Receptor Gamma and AMP-activated Protein Kinase [132]. Additionally, Ashour et al. (2012) and Mohanty et al. (2008) have documented its antioxidant activities and protective effects against cardiotoxicity in preclinical settings [133,134]. Guo & Rezaei (2024) [135] and Tiwari et al. (2021) [136] have also noted the adaptogenic and health-promoting effects of *W. somnifera* root extracts, particularly in enhancing cardiorespiratory endurance in athletes. These insights collectively affirm the therapeutic versatility of *W. somnifera*, particularly in cardiovascular health, which is supported by traditional uses and modern pharmacological research [135,136]. These outcomes appear promising, yet they differ based on the dose, extract form, and duration of administration (see Section 7 for a comparative analysis).

Recent studies, such as those by Paul et al. (2021) and Behl et al. (2020), have demonstrated that *W. somnifera* extracts effectively mitigate doxorubicin-induced cardiotoxicity by reducing oxidative stress, inflammation, and apoptosis in rodent models [122,132]. Multiple randomized controlled trials have consistently demonstrated the stress-relieving, cardioprotective, and adaptogenic benefits of ashwagandha. Clinical studies have shown significant reductions in anxiety and stress, along with improvements in cardiorespiratory endurance and overall cognitive function [130,137,138].

It is important to recognize that rodent models of doxorubicin-induced cardiotoxicity, though commonly used, do not fully replicate the cardiotoxic effects observed in humans. Studies using rabbit or other larger animal models that more closely mimic human cardiac physiology can offer greater translational relevance and should be considered in future research to confirm the cardioprotective effects observed in rodent studies [122,132].

Within cardiology, the described cardioprotective properties of *W. somnifera* could offer adjunctive therapeutic potential rather than being standalone treatments. Specifically, extracts of *W. somnifera* might help mitigate cardiac toxicity induced by anthracyclines like doxorubicin, potentially reducing adverse cardiac events in cancer patients. Furthermore, its beneficial effects might support conventional antihypertensive treatment by improving endothelial function, attenuating oxidative stress, and suppressing inflammatory pathways. However, it must be emphasized that these extracts are not intended to replace established pharmaceutical therapies. Further rigorous clinical trials and pharmacokinetic evaluations are essential to validate these applications, ensuring safety, clinical effectiveness, and compatibility with existing cardiovascular therapies (Table 4) [122,132].

### 9.4. Antibacterial Effect

*Withania somnifera* is an important MP renowned for its rich phytochemical composition, which significantly contributes to its antimicrobial properties. Its phytochemical arsenal includes withanolides, alkaloids such as witanin, somniferin, and tropin, flavonoids like 3-O-rutinoside and quercetin derivatives, withanolid glycosides (sitoindoside IX and X), steroidal saponins, coumarins, sterols, and various other metabolites [14,126]. These compounds are implicated in a multifaceted antimicrobial mechanism that includes immunomodulatory effects, cytotoxicity, and gene silencing [124,126]. Research has demonstrated *W. somnifera*’s efficacy against a broad spectrum of pathogens, including *methicillin-resistant S. aureus*, *Enterococcus* spp., and various gram-negative bacteria such as *E. coli*, *Proteus mirabilis*, *P. aeruginosa*, *Salmonella typhi* (*S. typhi*), *Citrobacter freundii*, and *K. pneumoniae* [126]. Notably, it is particularly effective against *S. typhi*, both in vitro and in animal models [122,126].

Recent studies have further substantiated the antimicrobial prowess of *W. somnifera.* Owais et al. (2005) found that both aqueous and alcoholic extracts of *W. somnifera* roots and leaves possessed strong antibacterial activity against *Salmonella typhimurium* and enhanced the survival rates in Balb/C mice with *salmonellosis* [139]. Singh and Kumar (2011) demonstrated that the flavonoids from various parts of *W. somnifera* exhibited significant antimicrobial activities against several pathogens, including *C. albicans* and *S. aureus* [19]. Alam et al. (2012) reported the antioxidant and antibacterial activities of methanolic extracts from the leaves, fruits, and roots of *W. somnifera*, with leaf extracts showing the highest activity against *S. typhi* [18]. Lastly, El-Hefny et al. (2020) explored the antibacterial and antifungal activities of wood treated with *W. somnifera* fruit extract, showcasing its potential to inhibit plant pathogens [140]. These findings underscore *W. somnifera*’s significant potential as a source of natural antimicrobial compounds for various therapeutic applications. It enhances immune reactivity, induces cytotoxic effects, and participates in gene silencing, contributing to its overall antimicrobial efficacy (Table 4).

While antimicrobial properties are frequently reported, differences in extract concentration, target organisms, and assay methods contribute to variability in outcomes, as discussed in Section 7.

**Table 4:** Therapeutic Effects of *Withania somnifera*: Antidiabetic, Cardioprotective, and Antibacterial Properties in Various Experiments.

**Antidiabetic**						
Scientific name	Trial type	Model or method	Used Part or form	Dose and duration	Therapeutic effect	Reference
*Withania somnifera*	In vivo	Diabetic rat model	Withaferin A	-	Controlled Type 1 diabetes through Nrf2/NFκB signaling modulation.	[126,129]
	In vivo	Diabetic patients	Ashwagandha root powder	-	Improved lipid profiles, reduced body weight, and regulated blood pressure in diabetic patients.	[130,131]
	In vivo	Rat models	Aqueous extracts	200, 400 mg/kg for 5 days	Lowered blood glucose and HbA1c levels; anti-inflammatory and anti-glycating effects.	[122]
	In vitro	Cellular assays	Anti-adipogenic withanolides	-	Inhibited preadipocyte differentiation into adipocytes, potentially reducing obesity-related disorders.	[17]
**Cardioprotective**						
	In vivo	Male Wistar rats	Standardized *W. somnifera* extracts	300 mg/kg	Prevented doxorubicin-induced cardiotoxicity.	[141]
	In vivo	Rat model	*W. somnifera* extracts	1 mg/kg	Reduced myocardial necrosis and oxidative stress.	[17]
	In vivo	Rat model	*W. somnifera* extract	-	Protected against doxorubicin cardiotoxicity; improved antioxidant status.	[122]
	In vitro	Cellular assays	Withaferin A	-	Activation of protective Nrf2 pathway, anti-apoptotic effects.	[126]
	In vivo	Rat model	Methanolic extract	-	Improved oxidative stress markers, reduced cardiac damage.	[133]
	In vivo	Wistar rats with IR injury	Oral *Withania somnifera*	50 mg/kg/day for 1 month	Reduced myocardial injury via antioxidant mechanisms.	[134]
	In vitro and In vivo	Various models (e.g., cancer, neurodegeneration)	Withaferin A	-	Multi-target therapeutic effects, including cardioprotection via multiple pathways.	[132]
	In vivo	Fifty healthy athletic adults	Ashwagandha root extract capsules	300 mg, twice daily for 8 weeks	Enhanced cardiorespiratory endurance, improved stress recovery, and antioxidant levels.	[136]
**Antibacterial**						
	In vitro	Disc diffusion method	Zinc nanoparticles (ZnONPs) from extracts	-	Antibacterial activity against *S. aureus*, *Klebsiella pneumoniae*, and *E. coli.*	[142]
	In vitro	Antimicrobial assays	Aqueous and alcoholic extracts	-	Activity against *Salmonella typhimurium* and other pathogens like *S. aureus* and *C. albicans*.	[139]
	In vivo	Balb/C mice model	Leaf and root extracts	-	Enhanced survival in *salmonellosis.*	[139]
	In vitro	Antimicrobial assays	Methanolic extracts	-	Highest antibacterial activity (leaf extracts) against *S. typhi.*	[18]
	In vitro	Antimicrobial protein assays	Flavonoids from extracts	-	Effective against methicillin-resistant *S. aureus*, Enterococcus spp., and Gram-negative bacteria like *E. coli*, *K. pneumoniae*, and *P. mirabilis.*	[122,126]
	In vitro	Antibacterial/fungal plant pathogen assays	Fruit extract	-	Antibacterial and antifungal activities against plant pathogens.	[140]

*W. somnifera*: *Withania somnifera*, DOX: Doxorubicin, Nrf2: Nuclear factor erythroid 2–related factor 2, NFκB: Nuclear factor kappa-light-chain-enhancer of activated B cells, IR: Ischemia–reperfusion, ZnONPs: Zinc oxide nanoparticles, *S. aureus*: *Staphylococcus aureus*, *E. coli*: *Escherichia coli*, *K. pneumoniae*: *Klebsiella pneumoniae*, *S. typhi*: *Salmonella typhi*, *C. albicans*, *Candida albicans*, HbA1c: Hemoglobin A1c, MCV—Mean corpuscular volume.

## 10. Comparative Evaluation and Contradictions

Despite the promising outcomes discussed throughout this review, contradictory findings are evident across several studies. For example, *S. lycopersicum* extracts have shown hypoglycemic effects in animal models at 90 mg/kg, yet human trials reported limited changes in fasting glucose [16,82]. Likewise, while *W. somnifera* demonstrated cardioprotective effects in rodent models at certain doses, its efficacy varied depending on the extract preparation, standardization, and experimental duration.

These discrepancies may arise from differences in study designs, including sample size, animal model, route of administration, and dosage variability. Furthermore, some studies lack sufficient detail on extract standardization or fail to test optimal dosing ranges. This highlights the need for harmonized protocols, dose-optimization trials, and head-to-head comparisons across models to validate efficacy and minimize conflicting evidence (Table 5 and Table 6).

**Table 5:** Comparative Bioactive Compounds per Plant with Pharmacological Actions and References.

Plant	Bioactive Compounds and Actions	References
*Phoenix dactylifera*	Flavonoids, phenolic acids, saponins, tannins—antioxidant, hypoglycemic, anti-inflammatory, cardioprotective	[13,51,59]
*Solanum lycopersicum*	Lycopene, β-carotene, polyphenols, vitamins C and E—antioxidant, antihypertensive, antidiabetic, antibacterial	[16,72,95]
*Trigonella foenum-graecum*	*Trigonelline*, diosgenin, 4-hydroxyisoleucine, galactomannan, saponins—insulin sensitizer, hypolipidemic, antibacterial	[15,64,110]
*Withania somnifera*	*Withanolides*, alkaloids (withanine), flavonoids, sitoindosides—adaptogenic, antidiabetic, cardioprotective, antimicrobial	[14,19,122]

**Table 6:** Comparative Summary of Traditional Uses, Bioactive Compounds, and Modern Applications.

Medicinal Plant	Traditional Uses	Key Bioactive Compounds	Modern Applications
*Phoenix dactylifera* (Date Palm)	Management of diabetes, hypertension, immune enhancement, nutritional support, and anemia treatment.	Flavonoids, phenolic acids, saponins, tannins	Antidiabetic, cardioprotective, antimicrobial activities; functional foods; dietary supplements; skin-care products.
*Solanum lycopersicum* (Tomato)	Antimicrobial, wound healing, gastrointestinal and skin disorders; cardiovascular support.	Lycopene, β-carotene, polyphenols, vitamins C and E	Antioxidant-rich functional foods, potential antidiabetic and cardiovascular supplements, and topical antimicrobial products.
*Trigonella foenum-graecum* (Fenugreek)	Diabetes management, cholesterol reduction, gastrointestinal health improvement, and anti-inflammatory use.	Trigonelline, diosgenin, galactomannans, 4-hydroxyisoleucine, saponins	Supplements for diabetes and hypercholesterolemia; antimicrobial agents; dietary fiber-enriched functional foods; anti-inflammatory preparations.
*Withania somnifera* (Ashwagandha)	Stress relief, immune enhancement, diabetes management, neurological and cardiovascular health, and anti-inflammatory treatments.	Withanolides, alkaloids (withanine), flavonoids, steroidal saponins	Adaptogenic supplements, antidiabetic and cardioprotective therapies, antimicrobial and neuroprotective formulations, functional beverages, and food products.

It is essential to clarify that the medicinal plant extracts discussed in this review are not proposed as direct substitutes for insulin treatment or other clinically established diabetic therapies. Rather, these plant-derived bioactive compounds are considered complementary or adjunctive therapies that may support conventional treatment approaches. While evidence suggests potential benefits in improving insulin sensitivity, glucose metabolism, and glycemic control, these natural compounds should not replace insulin or prescribed diabetes medications without thorough clinical validation and direct recommendations from healthcare professionals. Further clinical trials and rigorous safety evaluations are needed before any definitive conclusions or recommendations regarding insulin substitution can be made [15,111,143].

Moreover, it is imperative to emphasize that this review does not advocate the use of plant extracts as direct substitutes for conventional antibiotics in the treatment of systemic bacterial infections. Rather, these plant-derived bioactive compounds are presented primarily for their potential utility as complementary or adjunct therapies or as sources for the identification and subsequent development of novel antimicrobial agents. Effective therapeutic use of these compounds in systemic infections would necessitate appropriate pharmaceutical formulations, comprehensive pharmacokinetic assessments, and rigorous clinical validation. Present research predominantly addresses topical or localized applications, aiming at the preliminary screening and characterization of active compounds with antimicrobial potential. Consequently, the practical applicability of these plant-derived extracts in systemic bacterial infections remains hypothetical at present and requires detailed future investigations through controlled clinical trials [1,2].

Plant extracts intended for the management of diabetic conditions could be utilized primarily as adjunctive or complementary therapies rather than standalone treatments. These extracts may function through various mechanisms, such as enhancing insulin sensitivity, improving glucose uptake in peripheral tissues, modulating pancreatic β-cell function, and exerting antioxidant and anti-inflammatory effects, all of which are beneficial in diabetes management. Administration methods could vary, including oral consumption in standardized capsules or tablets, integration into functional foods, or as components in nutraceutical formulations. Before clinical recommendations can be made, it is critical that these extracts undergo rigorous pharmacokinetic evaluations, dose-optimization studies, and controlled clinical trials to confirm efficacy, safety, optimal dosing regimens, and possible interactions with conventional diabetic medications [15,111,143].

## 11. Comparative Analysis with Traditional Chinese Medicine (TCM)

To further contextualize these findings within a broader global perspective, the following section provides a comparative analysis with Traditional Chinese Medicine (TCM). While this review primarily focuses on medicinal plants traditionally utilized in the Middle East, it is valuable to briefly compare these findings with perspectives from Traditional Chinese Medicine (TCM). TCM is a systematically developed medical system extensively using medicinal plants characterized by distinctive phytochemical profiles and therapeutic effects [144,145]. Shared therapeutic objectives between Middle Eastern plants and TCM herbs include managing diabetes, cardiovascular diseases, and microbial infections. Notably, TCM herbs such as *Panax ginseng*, *Astragalus membranaceus*, and *Lycium barbarum* (Goji berry) demonstrate significant antidiabetic, cardioprotective, and antimicrobial activities, respectively [137,138,146]. Similar to Middle Eastern medicinal plants, TCM herbs contain bioactive compounds, including saponins, flavonoids, alkaloids, and polysaccharides, that modulate oxidative stress, inflammation, and metabolic pathways [147,148].

Ashwagandha, “Indian ginseng,” is prominently featured in Ayurvedic medicine and has been recognized within TCM frameworks for its adaptogenic properties, stress relief, and immune-enhancing effects [29,121]. Fenugreek (*Trigonella foenum-graecum*) is similarly used in TCM to dispel cold, improve circulation, and alleviate pain, highlighting a convergence in therapeutic applications across traditional medical practices [30,149].

However, distinctive features between the traditions persist. TCM emphasizes the synergistic interaction of multiple herbal components within complex herbal formulas, whereas Middle Eastern practices often highlight potent effects derived from single or limited-combination herbal applications [150]. Additionally, diagnostic patterns in TCM focus on holistic assessments through symptoms and signs such as tongue diagnosis, pulse diagnosis, and an understanding of Qi (vital energy) imbalance [151]. In contrast, Middle Eastern herbal medicine typically centers more directly on symptom-targeted interventions without explicit systematic diagnostic frameworks analogous to TCM [152].

Acknowledging both similarities and differences between these medicinal traditions enriches our understanding and supports broader integrative therapeutic strategies, emphasizing the global significance of medicinal plants in contemporary healthcare.

## 12. Safety Profiles and Toxic Effects of Medicinal Plants

While MPs such as date palm (*P. dactylifera*), fenugreek (*T. foenum-graecum*), tomato (*S. lycopersicum*), and ashwagandha (*W. somnifera*) offer significant therapeutic benefits, it is essential to acknowledge their safety profiles and potential toxic effects. Traditional texts and modern scientific literature indicate that improper use, excessive dosage, or prolonged administration of certain medicinal plants may lead to adverse reactions. For instance, fenugreek has been associated with gastrointestinal disturbances and hypoglycemia at high doses [153,154]. Ashwagandha, although generally safe at therapeutic doses, may cause gastrointestinal symptoms or interact negatively with sedatives and immunosuppressants if not used appropriately [29,155]. Tomato plants contain glycoalkaloids like tomatine, which can exhibit toxic effects at elevated concentrations, causing symptoms such as nausea, diarrhea, and abdominal pain [156]. While date palm fruits are broadly recognized as safe, excessive consumption of dates, particularly by diabetic patients, may result in elevated blood glucose levels due to their high sugar content [13]. Therefore, careful consideration of dosage, plant part used, preparation methods, and possible herb/drug interactions is crucial to ensuring safety and efficacy in traditional and modern medicinal contexts.

## 13. Conclusions and Future Perspectives

This review systematically evaluates four widely used medicinal plants—*P. dactylifera*, *S. lycopersicum*, *T. foenum-graecum*, and *W. somnifera*—in terms of their antidiabetic, cardioprotective, and antimicrobial properties. By addressing the research question, we compared the bioactive mechanisms of these species and assessed their therapeutic relevance across traditional and modern contexts.

The findings indicate that all four plants contain rich phytochemical profiles, particularly flavonoids, phenolic compounds, alkaloids, and saponins, which are linked to key biological activities such as enzyme inhibition, anti-inflammatory signaling, and oxidative stress modulation. Although the evidence supports their biomedical potential, variability in extract composition, dosage, and experimental design limits direct comparisons and universal application.

One limitation of this review lies in the heterogeneity of included studies. Most are preclinical, with few standardized clinical trials. Additionally, the diversity of extract types and inconsistency in dose reporting limit our ability to establish definitive therapeutic windows.

Future research should focus on:

Standardized clinical trials to validate the safety and efficacy of these plants in human populations;

Dose optimization and toxicity assessment using harmonized experimental protocols;

Exploring synergistic effects of plant combinations or plant/drug interactions;

Biotechnological approaches to enhance bioactive compound yield and stability.

Beyond their pharmacological interest, medicinal and aromatic plants also possess socio-cultural and ecological value. In many regions—such as the Middle East, South Asia, and the Mediterranean—the knowledge and use of these plants are integral to cultural identity, traditional practices, and rural livelihoods. While this review does not advocate the clinical use of unstandardized plant mixtures, we acknowledge that preserving ethnobotanical knowledge contributes to biodiversity conservation and sustainable development. When supported by scientific validation and regulatory oversight, such practices may play a role in community-based health systems and ecotourism. As discussed by Capucho et al. (2023), valuing these resources through responsible cultivation and educational initiatives aligns with global sustainability goals, including cultural heritage preservation and environmental stewardship [157].

In summary, this review highlights the complementary therapeutic potential of *P. dactylifera*, *S. lycopersicum*, *T. foenum-graecum*, and *W. somnifera*, focusing on their bioactive compounds and mechanistic actions. While these plant-derived agents are not positioned as alternatives to standard medical care, their promising pharmacological profiles justify further investigation in adjunctive therapy development and bioprospecting. Future research should focus on clinical validation, dosage optimization, and safety profiling to facilitate their integration into evidence-based healthcare strategies.

## Data Availability

No new data were created or analyzed in this study. Data sharing is not applicable to this article.
